# Haptoglobin 2-2 genotype is associated with increased risk of cardiovascular disease in patients with rheumatoid arthritis: a matched case-control study

**DOI:** 10.3389/fmed.2024.1442858

**Published:** 2024-12-17

**Authors:** Chuanhui Xu, Lay Wai Khin, Hui Zhen Tam, Liuh Ling Goh, Ee Tzun Koh, Rinkoo Dalan, Khai Pang Leong

**Affiliations:** ^1^Department of Rheumatology, Allergy and Immunology, Tan Tock Seng Hospital, Singapore, Singapore; ^2^Lee Kong Chian School of Medicine, Nanyang Technological University, Singapore, Singapore; ^3^Clinical Research & Innovation Office, Tan Tock Seng Hospital, Singapore, Singapore; ^4^Molecular Diagnostic Laboratory, Tan Tock Seng Hospital, Singapore, Singapore; ^5^Department of Endocrinology, Tan Tock Seng Hospital, Singapore, Singapore

**Keywords:** rheumatoid arthritis, cardiovascular disease, *Haptoglobin* genotypes, inflammation, personalized medicine

## Abstract

**Introduction:**

Traditional risk factors do not fully explain the increased risk of cardiovascular disease (CVD) in patients with rheumatoid arthritis (RA). The *Haptoglobin (Hp)* 2-2 genotype confers a lower anti-oxidant and higher inflammatory effect on the vasculature compared to the non-*Hp* 2-2 genotype. This study investigates the association of the *Hp* genotype with CVD in patients with RA.

**Methods:**

Data from 69 RA patients with CVD and 207 sex- and ethnicity-matched RA patients without CVD, collected from 1 January 2000 to 31 December 2020, were retrieved from the Tan Tock Seng Hospital RA Registry. CVD was examined against demographics, clinical and laboratory variables in univariate models. Associations between the *Hp* genotypes and CVD were analyzed using conditional logistic regression.

**Results:**

We studied 276 patients (65.2% female, 82.6% Chinese, median age 60.9 years). Most participants were in low disease activity or remission (79.3%). The *Hp* 2-2 genotype was present in 49.6% (137/276). In the group with CVD, the prevalence of the *Hp* 2-2 genotype was 50.9% (29/57) in the Chinese, 100% (5/5) in the Indians, and 28.6% (2/7) in the Malays. In the non-CVD group, the respective prevalence was 46.8% (80/171), 66.7% (10/15), and 52.4% (11/21). In univariate analysis, the matched odds ratio (OR) of the *Hp* 2-2 genotype for CVD in RA was 1.34 [95% confidence interval (CI): 1.22–1.47; *p* < 0.001]. The *Hp* 2-2 genotype was significantly associated with CVD (adjusted matched OR: 1.13; 95% CI: 1.01–1.27; *p* = 0.033) in the multivariate logistic regression model after adjusting the confounding factors, including age, smoking, diabetes, hypertension, hyperlipidemia, anti-CCP autoantibodies, and disease activity.

**Conclusion:**

The *Hp* 2-2 genotype is associated with an increased risk of CVD in patients with RA in this multi-ethnic cohort.

## Introduction

Rheumatoid arthritis (RA) is an archetype of multi-systemic chronic inflammatory disease (1). Cardiovascular disease (CVD) is one of the major causes of mortality and morbidity of RA (1). The risk of CVD in RA is comparable to that conferred by diabetes mellitus (DM), after adjusting for the traditional risk factors (2). CVD disproportionately affects the young RA population (3). The European Alliance of Association for Rheumatology (EULAR) recommends raising the risk derived from standard algorithms by 1.5 for RA patients (4). However, it has been argued that this method does not reclassify into more appropriate risk categories since it does not address RA-specific risks.

Haptoglobin (Hp) is an acute phase reactant that prevents oxidative damage by binding oxygenated free hemoglobin (5). There are two alleles of the *Hp* gene, namely *Hp*1 and *Hp*2, and three genotypes, *Hp* 1-1, *Hp* 1-2 and *Hp* 2-2. The anti-oxidant effect of *Hp* 2-2 is inferior to the other two (5). *Hp* 2-2 is associated with an increased risk of CVD in patients with DM (6–9). *Hp* 2-2 is overexpressed in patients with a family history of RA (10) and systemic lupus erythematosus (SLE) (11, 12). High serum level of Hp is associated with inadequate response to methotrexate in RA (13). However, the role of *Hp* genotypes in reclassifying CVD risk in RA has not been investigated. This study aims to study the association of *Hp* genotypes with CVD in RA in a multi-ethnic Asian cohort.

## Materials and methods

### Patient’s clinical data and sample

The Tan Tock Seng Hospital (TTSH) RA Registry is a longitudinal multi-ethnic disease registry in Singapore inaugurated in 2001 (14, 15). RA patients fulfilled the 1987 American Rheumatism Association criteria or the 2010 American College of Rheumatology (ACR)/EULAR criteria (16, 17). The presence of CVD was reported by the attending physicians. We identified 69 patients with CVD in our Registry. We also selected sex- and ethnicity-matched RA patients without CVD in a ratio of one case to three controls. Biobanked DNA samples, serum samples, and matching clinical data were retrieved. The study was approved by the institutional review board (NHG DSRB reference number 2006/00011).

### Haptoglobin genotype and protein measurement

The *Hp* genotyping was performed using TaqMan-based real-time polymerase chain reaction (PCR) as previously described (18). Plasma haptoglobin was measured using the immunoturbidimetric method on the Beckman Coulter AU system.

### Statistics

The distribution of demographic and clinical characteristics was summarized using descriptive statistical methods. The normality of the data was assessed for continuous variables; mean (standard deviation, SD) or median (interquartile range, IQR) were used to summarise normally distributed or skewed data, respectively. Frequency and percentage were used to summarise the categorical variables.

Univariate and multivariate conditional logistic regression were performed to estimate the effect size of the covariate of interest, *Hp* 2-2 genotype, in the prediction of pre-specified clinical outcome (i.e., presence or absence of CVD event), because the study design was a gender and ethnicity matched case-control study (19, 20).

The pre-specified clinical outcome was the presence or absence of a CVD event (binary outcome), and the main covariate of interest was the *Hp* 2-2 genotype.

In our study, variables were initially selected for inclusion in the model if they were either theoretically relevant based on prior literature or demonstrated a bivariate association with the outcome at a significance level of *p* ≤ 0.10.

In the univariate variable selection stage, variables with a *p*-value ≤0.1 with odds ratios that exclude 1 were selected as preliminary predictors for inclusion in the multivariate model in order not to miss any potentially important clinical predictors. For the final multivariate conditional logistic regression model, statistical significance was defined by the conventional *p* ≤ 0.05, with odds ratios excluding 1.

In the multivariate conditional logistic regression model, a stepwise backward regression approach with robust variance estimation and frequency weighted analysis options were applied to account for the 1:3 matching (1 case:3 controls) in the study design.

During the backward stepwise process, covariates identified as potential confounders were included based on known associations with both the exposure and outcome variables with pre-determined *p*-value cutoff ≤0.10, as well as including variables exhibiting *p*-value ≤0.10 in the univariate variable selection stage. This approach was adopted to ensure that potentially significant clinical predictors were retained in the multivariate model.

Variables were then removed sequentially if they did not meet the final significance threshold of *p* < 0.05 with odds ratios excluding 1 after adjusting for other variables in the model.

To assess the model fit, the Hosmer–Lemeshow goodness-of-fit test was performed. The final model selection was based on the model with the lower deviance, defined by the −2×log-likelihood (−2LL) value, which is the better model. The final fitted model was chosen based on the −2×log-likelihood (−2LL) value with the number of significant clinically important variables in the model.

Effect sizes were presented as adjusted matched odds ratios (matched OR) with 95% CI.

Statistical significance was set at two-sided 5% level and all analyses were conducted using STATA 16.1.

## Results

### Clinical characteristics of patients with RA

This study included 276 patients, mostly female (65.2%) and of Chinese ethnicity (82.6%). There were 69 RA patients with CVD and 207 sex- and ethnicity-matched RA patients without CVD. The median age was 60.9 years [interquartile range (IQR): 53.8–68.0], and a quarter of the patients had a history of smoking (Table 1). The prevalences of diabetes, hypertension, and hyperlipidemia were 14.5, 46.4, and 57.2%, respectively (Table 1). The median RA duration was 119.2 (IQR: 32.8–215.2) months, with high positivity for rheumatoid factor (84.8%) and anti-cyclic citrullinated peptides (anti-CCP) autoantibodies (80.1%), and most were in remission or low disease activity (79.3%) (Table 1).

**Table 1:** Baseline characteristics and univariate analyses of the association between baseline characteristics and events of CVD in patients with RA.

	All	No CVD	CVD	Univariate		
	*n* = 276	*n* = 207	*n* = 69	Matched OR	95% CI	*p*
Demographic						
Age (years), median (IQR)	60.9 (53.8–68.0)	59.8 (52.4–66.5)	66.6 (59.3–74.0)	1.08	1.08–1.09	**<0.001**
Female, *n* (%)	180 (65.2)	135 (65.2)	45 (65.2)			
Ethnicity						
Chinese, *n* (%)	228 (82.6)	171 (82.6)	57 (82.6)			
Malay, *n* (%)	28 (10.1)	21 (10.1)	7 (10.1)			
Indian, *n* (%)	20 (7.3)	15 (7.3)	5 (7.3)			
Smoking status						
Ever, *n* (%)	69 (25.0)	52 (25.2)	17 (24.6)	1.00	0.88–1.13	0.961
Clinical risk factors						
Disease duration (months)	119.2 (32.8–215.2)	114.9 (31.3–207.0)	121.1 (39.3–251.8)	1.10	1.07–1.14	**<0.001**
Diabetes, *n* (%)	40 (14.5)	23 (11.2)	17 (25.0)	2.54	2.25–2.87	**<0.001**
Hypertension, *n* (%)	128 (46.4)	77 (37.4)	51 (75.0)	5.76	5.14–6.46	**<0.001**
Hyperlipidemia, *n* (%)	158 (57.2)	105 (51.0)	53 (77.9)	4.07	3.62–4.59	**<0.001**
Positive RF, *n* (%)	234 (84.8)	173 (84.8)	61 (89.7)	1.19	1.04–1.37	**0.014**
Positive anti-CCP, *n* (%)	221 (80.1)	161 (77.8)	60 (87.0)	2.39	2.07–2.77	**<0.001**
DAS-28						
Remission/low disease activity, *n* (%)	219 (79.3)	165 (80.1)	54 (78.3)	ref	ref	**<0.001**
Moderate/severe disease activity, *n* (%)	56 (20.3)	41 (19.9)	15 (21.7)	1.33	1.17–1.50	
Cumulative prednisolone (g), median (IQR)	4.1 (1.1–1.2)	3.6 (0.9–1.1)	4.9 (2.0–14.9)	1.0007	1.0003–1.0011	**<0.001**
Genotype						
*Hp* 2-2 Genotype, n (%)	137 (49.6)	101 (48.8)	36 (52.2)	1.34	1.22–1.47	**<0.001**

CVD, cardiovascular disease; RA, rheumatoid arthritis; *n*, number; OR, odds ratio; 95% CI, 95% confidence interval; RF, rheumatoid factor; CCP, cyclic-citrullinated peptide; DAS-28, disease activity score 28; ref, reference; IQR, interquartile range. Median (IQR) for continuous variables and *n* (%) for categorical variables were shown. Bold values indicates statistically significant.

### *Hp* 2-2 genotype in different populations

The overall prevalence of the *Hp* 2-2 genotype was 49.6% (137/276). We found that 47.8% (109/228) of Chinese, 46.4% (13/28) of Malay, and 75% (15/20) of Indian patients carry the *Hp* 2-2 genotype (Table 2). Among patients with CVD classified into the three ethnicities, 50.9% (29/57) of the Chinese, 28.6% (2/7) of the Malays, and 100% (5/5) of the Indians carry *Hp* 2-2 genotype (Table 2). Among the patients without CVD, there were 46.8% (80/171) Chinese, 52.4% (11/21) Malay, and 66.7% (10/15) Indian patients carrying the *Hp* 2-2 genotype (Table 2). The haptoglobin protein level was significantly lower in patients with the *Hp* 2-2 genotype (mean ± SD: 130.0 ± 66.3 mg/dL) than those with the non *Hp* 2-2 genotype (mean ± SD: 161.2 ± 82.8 mg/dL) (*p* < 0.001) (Table 2).

**Table 2:** Overall characteristics of patients based on *Hp* 2-2 genotype status.

	Non-Hp 2-2 (*n* = 139)		*p*	Hp 2-2 (*n* = 137)		*p*
	Control (*n* = 106)	Case (*n* = 33)		Control (*n* = 101)	Case (*n* = 36)	
Age (years) median (IQR)	59.9 (53.0–66.9)	66.7 (59.3–73.9)	**<0.001**	59.7 (52.3–66.1)	66.0 (59.1–75.0)	**<0.001**
Female, *n* (%)	70 (66.0)	19 (57.6)	—	65 (64.4)	26 (72.2)	—
Ethnicity						
Chinese, *n* (%)	91 (85.9)	28 (84.9)	—	80 (79.2)	29 (80.6)	—
Malay, *n* (%)	10 (9.4)	5 (15.2)	—	11 (10.9)	2 (5.6)	—
Indian, *n* (%)	5 (4.7)	0 (0)	—	10 (9.9)	5 (13.9)	—
Smoking						
Ever, *n* (%)	27 (25.5)	10 (30.3)	**0.011**	25 (25.0)	7 (19.4)	**0.011**
Diabetes, *n* (%)	11 (10.4)	8 (24.2)	**<0.001**	12 (12.0)	9 (25.7)	**<0.001**
Hypertension, *n* (%)	36 (34.0)	26 (78.8)	**<0.001**	41 (41.0)	25 (71.4)	**<0.001**
Hyperlipidemia, *n* (%)	54 (50.9)	26 (78.8)	**<0.001**	51 (51.0)	27 (77.1)	**<0.001**
Haptoglobin (mg/dL), mean (SD)	161.2 (82.8)		—	130.0 (66.3)		**<0.001**
Haptoglobin (mg/dL), mean (SD)	161.0 (85.0)	162.0 (76.4)	0.316	126.3 (64.1)	140.2 (72.2)	**<0.001**
DAS-28						
Remission/low disease activity, *n* (%)	83 (78.3)	27 (81.8)	**0.001**	82 (82.0)	27 (75.0)	**<0.001**
Moderate/severe disease activity, *n* (%)	23 (21.7)	6 (18.2)		18 (18.0)	9 (25.0)	
Positive RF, *n* (%)	90 (85.7)	29 (87.9)	**0.034**	83 (83.8)	32 (91.4)	**<0.001**
Positive anti-CCP, *n* (%)	85 (80.2)	30 (90.9)	**0.001**	76 (75.3)	30 (83.3)	**0.037**
Disease duration (months), median (IQR)	88.9 (20.4–165.0)	106.4 (63.2–165.1)	**0.001**	106.4 (31.2-224.6)	156.8 (33.3–244.7)	**0.014**
Cumulative prednisolone (g), median (IQR)	3.4 (0.9–8.6)	5.4 (3.2–18.4)	**0.001**	3.8 (0.9–13.2)	4.5 (1.7–13.6)	**<0.001**

*n*, number; RF, rheumatoid factor; CCP, cyclic-citrullinated peptide; DAS-28, disease activity score 28. Mean (SD, standard deviation) or median (IQR, interquartile range) for continuous variables and *n* (%) for categorical variables were shown. Bold values indicates statistically significant.

### Univariate analysis of risk factors for CVD

In univariate analysis, traditional clinical risk factors significantly associated with CVD events were age (66.6 vs. 59.8 years in CVD and control group respectively, *p* < 0.001), diabetes (25.0% vs. 11.2%, OR 2.54, 95% CI 2.25–2.87, *p* < 0.001), hypertension (75% vs. 37.4%, OR 5.76, 95% CI 5.14–6.46, *p* < 0.001), and hyperlipidemia (77.9% vs. 51.0%, OR 4.07, 95% CI 3.62–4.59, *p* < 0.001) (Table 1). RA-specific factors associated with CVD events include disease duration (median 118.6 vs. 100.3 months in CVD and control group respectively, *p* < 0.001), rheumatoid factor and anti-CCP autoantibodies positivity (OR 1.19, *p* = 0.0135 and OR 2.39, *p* < 0.001, respectively), moderate and severe disease activity (OR = 1.33, 95% CI 1.17–1.50, *p* < 0.001), and higher cumulative dose of prednisolone (4.9 g vs. 3.6 g in CVD and control groups respectively, *p* < 0.001) (Table 1).

The prevalence of the *Hp* 2-2 genotype was higher in the CVD group compared to controls (52.2% vs. 48.8%), with a matched odds ratio of 1.34 (95% CI 1.22–1.47, *p* < 0.001) (Table 1).

### Multivariate analysis of risk factors for CVD

In multivariate analysis, after adjusting for age, smoking, diabetes, hypertension, hyperlipidemia, anti-CCP autoantibodies, and disease activity, the *Hp* 2-2 genotype remained independently associated with CVD (adjusted matched OR 1.13, 95% CI 1.01–1.27, *p* = 0.033) (Table 3). Other statistically significant associations with CVD events include age (adjusted matched OR 1.06, *p* < 0.001), smoking (adjusted matched OR 1.43, *p* < 0.001), diabetes (adjusted matched OR 1.21, *p* = 0.013), hypertension (adjusted matched OR 2.78, *p* < 0.001), hyperlipidemia (adjusted matched OR 2.77, *p* < 0.001), the presence of anti-CCP autoantibodies (adjusted matched OR 3.27, *p* < 0.001), and moderate/severe disease activity (adjusted matched OR 2.21, *p* < 0.001) (Table 3).

**Table 3:** Multivariate analyses of the association between *Hp* genotype and events of CVD in patients with RA.

	Adjusted matched OR	95% CI	*p*
*Hp* 2-2 genotype	1.13	1.01–1.27	**0.033**
Age	1.06	1.05–1.07	**<0.001**
Smoking	1.43	1.22–1.68	**<0.001**
Diabetes	1.21	1.04–1.42	**0.013**
Hypertension	2.78	2.42–3.19	**<0.001**
Hyperlipidemia	2.77	2.43–3.14	**<0.001**
Positive anti-CCP	3.27	2.80–3.82	**<0.001**
DAS-28			
Remission/low disease activity	reference	—	**<0.001**
Moderate/severe disease activity	2.21	1.86–2.61	

Hp, haptoglobin; RA, rheumatoid arthritis; OR, odds ratio; 95% CI, 95% confidence interval; CCP, cyclic-citrullinated peptide; DAS-28, disease activity score 28; CVD, cardiovascular disease. Bold values indicates statistically significant.

## Discussion

Our study shows that the *Hp* 2-2 genotype is significantly associated with the risk of CVD in RA patients in a Singaporean multi-ethnic cohort, independent of traditional CVD risk factors. This suggests that the *Hp* 2-2 genotype could be a potential biomarker for more accurate CVD risk predication in RA patients.

We found that the prevalence of the *Hp* 2-2 genotype varies among different ethnic groups. In decreasing order of frequency, they are Indians (75.0%), Chinese (47.8%), and Malays (46.4%), which aligns with previous studies on diabetes in Singapore (18) and other countries (21). The prevalence of RA also varies among different ethnicities, with a higher prevalence in India compared to other Asian countries (22). This could be due to genetic and environmental factors (22, 23). Previous studies reported that the prevalence of the *Hp* 2-2 genotype was higher in patients with a family history of RA (10) and SLE (11, 12). The over-representation of Indian ethnicity (12.1%) in our multi-ethnic RA cohort (14), compared to 7.5% in the population (24), and the increased risk of CVD in the Indian population (25), suggest that the *Hp* 2-2 genotype could play a role in these differences. Strikingly, all Indian patients with CVD were *Hp* 2-2 genotype carriers in our study, although the number was low.

In our study, the protein level of Hp was not different between non-CVD and CVD patients within the non *Hp* 2-2 subgroup, but higher in CVD patients than non-CVD patients within the *Hp* 2-2 subgroup. The antioxidant function might be impaired despite higher protein levels in patients with *Hp* 2-2. Serum haptoglobin α2 (expressed by *Hp* 2–1 or *Hp* 2-2 genotype), with lower antioxidant capacity than haptoglobin α1 (expressed by *Hp* 1–1 genotype), was found in higher concentration in patients with SLE (11). The high baseline haptoglobin protein level predicted poor response to MTX, independent of the DAS 28 score, and inflammatory markers (13). Our findings suggest the *Hp* 2-2 genotype may result in impaired antioxidant functions, potentially leading to enhanced inflammation and a diminished response to methotrexate, thereby increasing CVD risk.

The association of the *Hp* 2-2 genotype with DM and CVD complications in DM is well documented (6–9). Our study shows that the *Hp* 2-2 genotype is an independent risk factor for CVD in RA patients, even after adjusting traditional risk factors, including DM. The link between the *Hp* 2-2 genotype and increased CVD risk in our RA cohort is consistent with findings in DM populations. This parallel suggests a common pathogenic mechanism in these chronic inflammatory conditions (26). Inflammation is a stronger predictor for CVD than LDL in this era of statin therapy (27). The impaired antioxidant function of Hp 2-2 might lead to chronic inflammation. Furthermore, the *Hp* 2-2 genotype is associated with the disease severity (28), and survival (29) in CVD. Therefore, antioxidant therapies could be investigated as a potential intervention to mitigate CVD complications in the RA population, as demonstrated in DM (9).

The burden of CVD in RA is comparable to that in DM (2). The traditional risk prediction model is not accurate in RA, even with the 1.5-time multiplier recommended by EULAR (4). *Hp* polymorphism has been extensively studied in patients with DM, and the *Hp* 2-2 genotype has shown the potential for refining the cardiovascular risk assessment (30, 31). Moreover, the predictive value of traditional risk factors, i.e., elevated glycosylated hemoglobin (HbA1c), is more pronounced among patients with the *Hp* 2-2 genotype (7, 32). Elevated homocysteine levels (33) and increased Carotid Intima-Media Thickness (CIMT) (34) are associated with increased CVD risk. Incorporating risks such as *Hp* 2-2, homocysteine levels, and CIMT into risk algorithms could enhance their predictive accuracy and enable precise risk stratification in RA, leading to timely implementation of optimal therapy and ultimately improving outcomes.

This study has a few strengths. First, a multi-ethnic Asian cohort allows for examining genetic risk factors across diverse populations. This is particularly relevant given the variability in the prevalence of the *Hp* 2-2 genotype among different ethnicities. Second, the study has 20-year follow-up, providing a broad dataset for analysis. Third, by matching controls to cases on important variables such as sex and ethnicity, the study design controlled for confounding factors. Limitations include the relatively small sample size, the retrospective CVD diagnosis potentially introducing selection bias, and the high proportion of likely post-menopausal women, which may elevate baseline CVD risk and affect generalizability. Additionally, it is known the medications use may affect the risk of CVD in RA (35). Therefore, the potential for bias due to unmatched medication use between the two groups warrants cautious interpretation.

Our study provides evidence for the *Hp* 2-2 genotype as an independent risk factor for CVD in patients with RA. Future research should focus on prospective validation of these findings in larger cohorts and explore the mechanistic pathways linking the *Hp* 2-2 genotype to CVD in RA. Furthermore, clinical trials assessing the efficacy of antioxidant therapies in reducing CVD risk in RA patients with the *Hp* 2-2 genotype would be a logical extension of this work.

## The TTSH Rheumatoid Arthritis Study Group

Andrea Ang, Angela Li-Huan Chan, Grace Yin Lai Chan, Madelynn Tsu-Li Chan, Faith Li-Ann Chia, Hiok Hee Chng, Choon Guan Chua, Hwee Siew Howe, Ee Tzun Koh, Li Wearn Koh, Kok Ooi Kong, Weng Giap Law, Samuel Lee Shang Ming, Khai Pang Leong, Tsui Yee Lian, Xin Rong Lim, Jess Mung Ee Loh, Mona Manghani, Justina Wei Lynn Tan, Sze-Chin Tan, Teck Choon Tan, Claire Teo Min-Li, Bernard Yu-Hor Thong, and Paula Permatasari Tjokrosaputro.

## Data Availability

The datasets used and/or analyzed for this study are available from the corresponding author upon reasonable request. Requests to access these datasets should be directed to Chuanhui Xu. E-mail: xuchuanhui2008@gmail.com.
